# Regulatory Interventions for Package Inserts and Patient Medication Information: A Systematic Review

**DOI:** 10.3390/healthcare14203464

**Published:** 2026-10-15

**Authors:** Wantong Yang, Linning Wang, Yun Lu, Feng Chang

**Affiliations:** School of International Pharmaceutical Business, China Pharmaceutical University, Nanjing 211198, China; 3224041289@stu.cpu.edu.cn (W.Y.);

**Keywords:** drug labeling, package insert, patient information leaflet, patient medication information, regulatory intervention, systematic review, readability, comprehension, medication safety, data

## Abstract

**Highlights:**

**What are the main findings?**

• Standardized organization, numerical presentation of risk, and performance-based user testing were generally associated with better information finding and comprehension.
• Evidence that these approaches change medication-taking behavior or reduce medication errors and adverse drug events was limited.

**Abstract:**

**Background/Objectives**: Package inserts and patient medication information aim to translate complex evidence into practical instructions for safer use. Regulators have standardized content, format, user testing, risk wording, and distribution, but effects along the pathway from readability to medication safety remain unclear. **Methods**: We searched PubMed/MEDLINE, Embase, Web of Science, Scopus, regulator websites, and citations up to 23 July 2026. Eligible reports evaluated a mandated or endorsed requirement, template, wording, testing process, distribution program, or named initiative. Two reviewers independently screened, assessed full texts, extracted data, and judged methodological quality, resolving disagreements by consensus. **Results**: Of 2817 records, 34 full texts were assessed, 6 excluded, and 28 reports (27 studies) included. Due to heterogeneity, we synthesized descriptively using an intervention–outcome framework. Standardized structure, user-centered redesign, numerical risk formats, and performance-based testing were generally associated with better information finding and comprehension. Verbal descriptors like “common” or “rare” produced higher perceived probabilities than numerical formats. Large US studies of Medication Guides and consumer information documented excessive length, high reading levels, incomplete objective comprehension, inconsistent distribution quality, and limited risk knowledge. Overall test performance sometimes masked failures in emergency sequencing, contraindications, or applicability. Evidence for adherence, errors, adverse events, and healthcare utilization remained sparse. **Conclusions**: The findings support prioritized core structure, plain language, numerical risk communication, target-user testing, delivery verification, accessible formats, and post-implementation monitoring. Benefits are best established for proximal communication outcomes; effects on real-world safety remain uncertain.

## 1. Introduction

Written information accompanies medicines in almost every contemporary regulatory system. It identifies the medicine, describes how it should be used, explains contraindications and interactions, and communicates the likelihood and seriousness of harm. For medicines used outside direct clinical supervision, the package insert, patient information leaflet (PIL), Drug Facts label, Medication Guide, consumer medication information (CMI), or proposed patient medication information (PMI) may be the only standardized information available when a patient decides whether and how to use a product. These documents are therefore not merely legal artifacts. They are risk-control tools whose performance depends on whether the intended reader can locate, interpret, remember, and act on essential content.

Regulatory systems distinguish the primary product label and package information required to identify and use a medicine from supplementary patient-facing materials that add plain-language counseling, risk-minimization instructions, or monitoring advice. The exact combination varies by jurisdiction and product risk, but all components should be reconciled with the approved product information. Leaflet creation therefore begins with a regulatory and medical review of the indication, dose, contraindications, interactions, warnings, and evidence of benefit and harm; writers then convert those requirements into a prioritized patient-facing structure, check legal completeness and safety-critical wording, test the draft with intended users, and control the approved version across print and digital channels. This process is a negotiated boundary between information that must be included for legal, medical, and safety reasons and information that can be added when it improves understanding without obscuring the core messages [1,2,3,4,5,6,7,8,9,10].

Regulators have attempted to improve this performance through several routes. European legislation established mandatory patient leaflets and later required consultation with target patient groups; associated guidance and the Quality Review of Documents template prescribe content, ordering, headings, and wording [1,2,3,4]. In the United States, the 1999 Drug Facts rule standardized the content and visual hierarchy of nonprescription labels, while label-comprehension guidance formalized performance testing for products considered for over-the-counter use [5,6]. Medication Guides and risk evaluation and mitigation strategies address selected prescription medicines [7], and the proposed one-page PMI rule seeks to replace fragmented pharmacy information with a consistent patient-facing document [8]. Health Canada introduced Plain Language Labelling requirements, and the United Kingdom has combined legal requirements with information-design guidance and a renewed modernization program [9,10].

**Figure 1:**
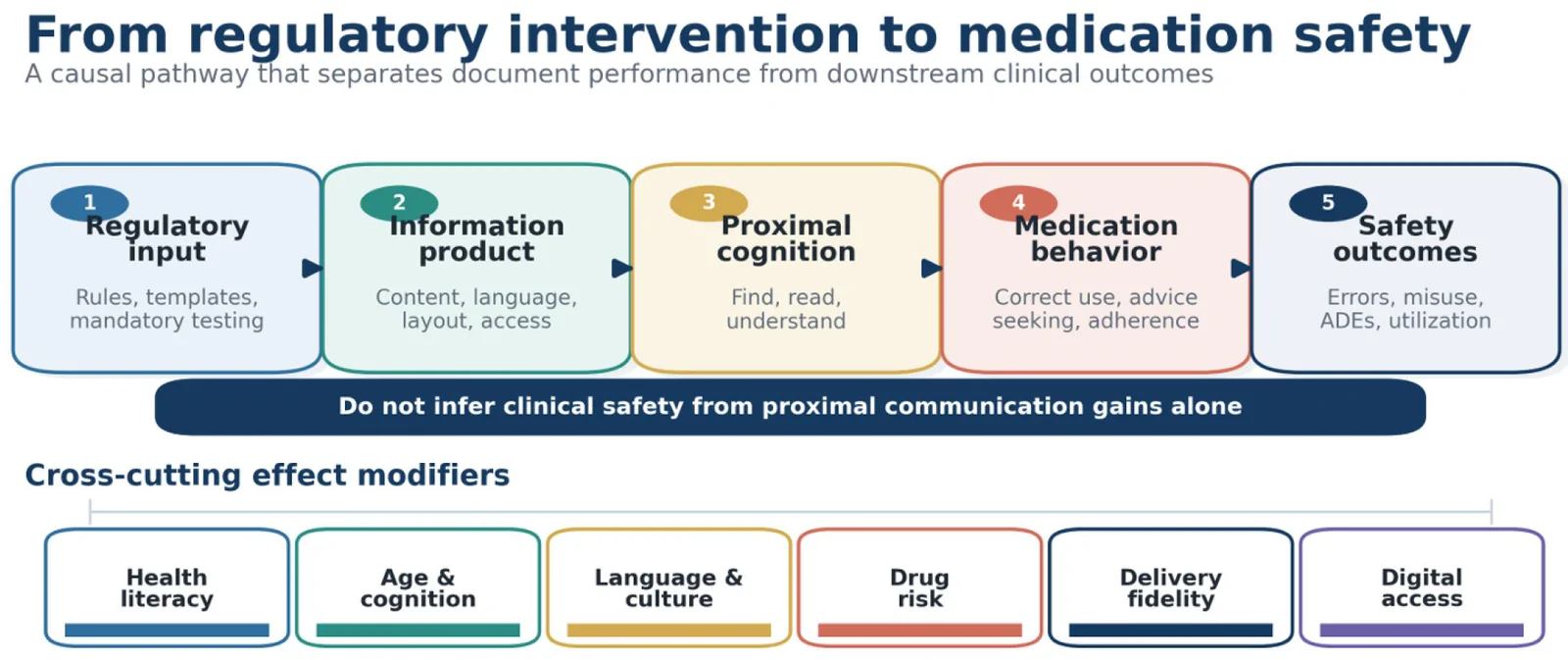
Conceptual pathway from regulatory intervention to medication safety. ADEs, Adverse drug events.

Moving from a regulatory rule to safer medicine use involves several steps. A new template may improve headings while leaving key concepts too technical. Plain language may lower the reading grade without improving understanding of contraindications. A label may perform well in an interview yet fail to reach patients at dispensing. Better risk knowledge may not change behavior, and changed behavior may not reduce preventable harm. Readability scores, user preferences, comprehension, and safety should therefore not be treated as interchangeable outcomes. Figure 1 separates regulatory inputs, changes to the information product, proximal cognitive outcomes, medication behavior, and distal safety outcomes, while recognizing health literacy, age, language, culture, drug risk, delivery fidelity, and digital access as effect modifiers.

Previous reviews summarized the readability of medicinal package leaflets and found that many documents used long sentences, technical vocabulary, small type, dense layout, or inconsistent organization, with readability varying across countries and products [11]. A later scoping review grouped factors that facilitate or hinder comprehension, including information design, prior knowledge, health literacy, language, age, and the complexity of contraindication and interaction messages [12]. Broader health-literacy research likewise demonstrates that reading ability, numeracy, and document design shape medication understanding [13,14]. These reviews largely addressed general readability or determinants of comprehension; they did not consistently trace an identifiable regulatory intervention through delivery, behavior, and safety outcomes. The present review therefore requires an explicit regulatory linkage and follows effects across a prespecified chain from the information product through cognition and behavior to medication safety. Its aims were to classify regulator-linked interventions, compare their effects on readability, usability, comprehension, risk perception, behavior, and safety, identify implementation and equity gaps, and translate the evidence into a reproducible regulatory cycle.

## 2. Materials and Methods

### 2.1. Review Design and Reporting

**Figure 2:**
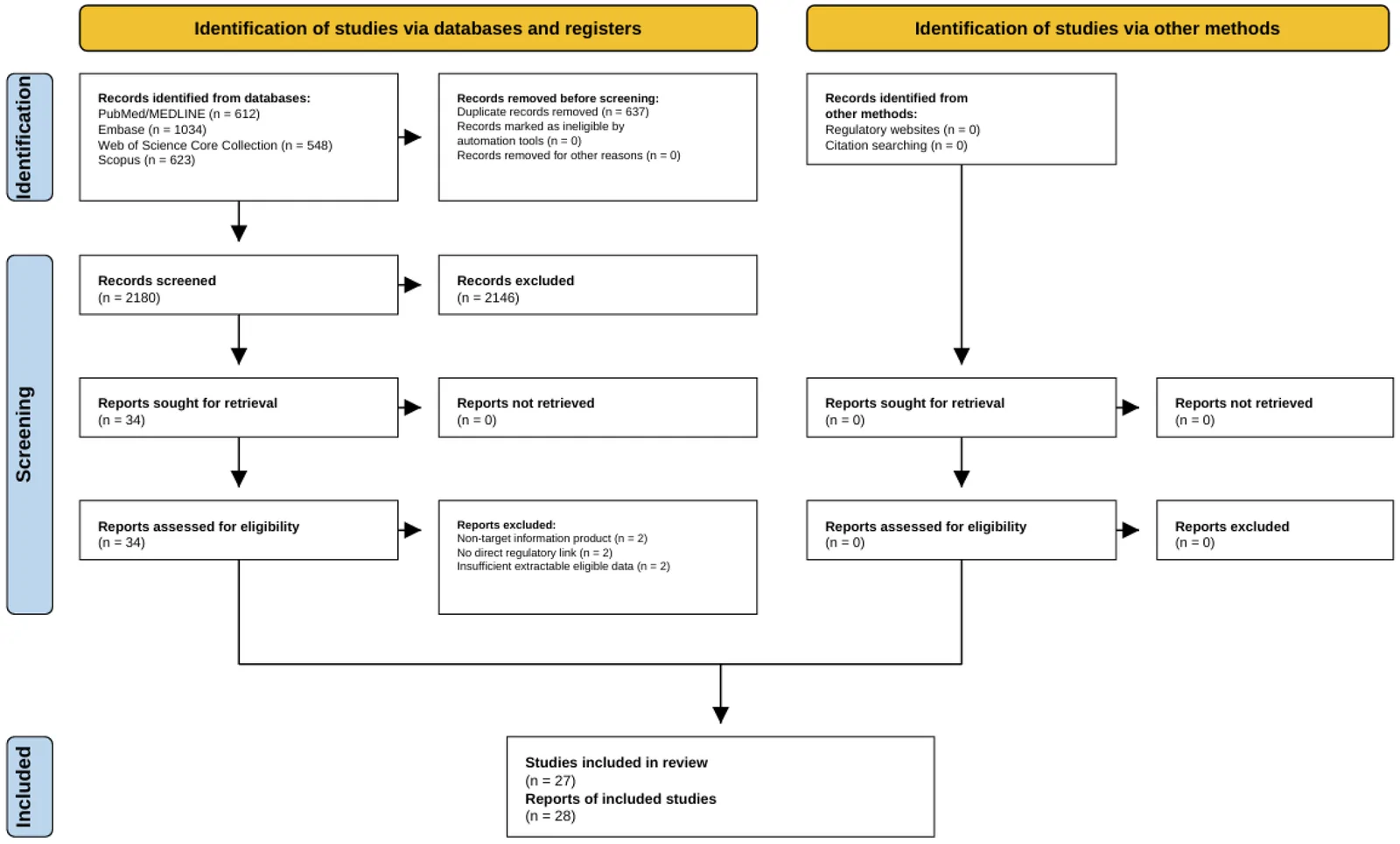
Evidence identification and selection.

This systematic review with narrative synthesis was reported with reference to PRISMA 2020 and the Synthesis Without Meta-analysis guideline [15,16]. A protocol was not prospectively registered. Before final screening, the reviewers specified the eligibility criteria, regulatory-linkage rule, extraction fields, outcome hierarchy, appraisal domains, and synthesis groups. The review question was organized around a causal pathway from regulatory intervention to information-product performance, proximal cognition, medication behavior, and clinical safety. This hierarchy prevented readability or preference outcomes from being interpreted as direct evidence of reduced medication harm. The completed PRISMA 2020 checklist is provided as Supplementary File S2, and the PRISMA flow diagram is shown in Figure 2. CONSORT was not applicable because this report is a systematic review rather than a randomized trial report.

### 2.2. Eligibility Criteria

Eligible reports evaluated an identifiable regulator-mandated requirement, regulator- endorsed guidance or template, standardized regulatory wording, performance-testing requirement, mandated information program, or an intervention explicitly developed to evaluate a named regulatory initiative. Eligible information products included package inserts, patient information leaflets (PILs), Drug Facts labels, Medication Guides, consumer medication information (CMI), patient medication information (PMI), and closely related patient-facing medicine risk materials. Comparative experiments, sequential user tests, label-comprehension studies, regulatory assessment reviews, implementation audits, and national surveys were eligible when the regulatory exposure was explicit. Investigator-developed leaflets or pictograms without a named regulatory program, legislation without outcome data, purely descriptive readability audits, general health education, clinician communication interventions, and non-medicine information sheets were excluded. Table 1 provides the operational definitions.

### 2.3. Information Sources and Search Strategy

PubMed/MEDLINE, Embase, Web of Science Core Collection, and Scopus were searched from inception through 23 July 2026, without date or language limits. The final saved searches identified 612 PubMed/MEDLINE records, 1034 Embase records, 548 Web of Science Core Collection records, and 623 Scopus records, for 2817 records before deduplication. FDA, EMA/European Commission, MHRA, and Health Canada websites were checked for policy context and additional empirical reports, and backward and forward citation searching was completed; neither source added records beyond the database yield. Dedicated grey-literature databases, such as OpenGrey, ProQuest Dissertations & Theses, and WHO ICTRP, were not searched. This choice may have increased the risk of publication and availability bias and is acknowledged as a limitation. Search concepts covered medicine information products, regulation or standardization, information design and user testing, comprehension and risk communication, implementation, behavior, and medication safety. Supplementary File S1 reports the final executed strategies, platforms, dates, and native hit counts. A targeted near-cutoff eligibility audit of potentially relevant 2025–2026 reports confirmed that excluded records lacked the prespecified regulatory linkage, an eligible comparative intervention, or reported results.

### 2.4. Study Selection, Extraction, and Independent Verification

Two reviewers independently screened 2180 deduplicated titles and abstracts and assessed 34 full-text reports. They independently extracted jurisdiction, regulatory context, study design, information product, intervention and comparator, sample, outcomes, numerical findings, effect direction, and limitations. Characteristics and extracted findings for all included reports are presented in Supplementary Table S1. Of the screened records, 2146 were excluded at title/abstract stage; six full-text reports were excluded with one primary report-level reason, and the reasons are listed in Supplementary Table S4; 28 reports were included. Reviewers independently verified citation details, companion-publication links, report-versus-study counts, sample sizes, and every numerical result used in the text, figures, and tables. Disagreements were resolved by discussion and reference to the source report. The archived review file retained consensus decisions but not reviewer-by-reviewer 2 × 2 decision tables, so Cohen’s kappa could not be calculated retrospectively; this limitation is reported explicitly below. Figure 2 summarizes selection.

### 2.5. Methodological Appraisal, Certainty, and Synthesis

Randomized experiments were assessed against RoB 2 domains; nonrandomized comparative evaluations were assessed using ROBINS-I concepts; and surveys, audits, diagnostic user tests, label-comprehension studies, and regulatory assessment reviews were assessed with prespecified design-adapted domains covering selection or allocation, comparability and confounding, outcome validity, missing data and selective reporting, and applicability. For cross-design presentation, overall judgments were harmonized as Low, Some concerns, or High; two reviewers independently assigned these judgments and reconciled differences, while applicability was reported separately from internal validity. Report-level methodological judgments are presented in Supplementary Table S2. Because interventions, outcome definitions, denominators, and designs were heterogeneous, meta-analysis was not appropriate. Synthesis followed prespecified intervention and outcome groups and considered effect direction, extractable magnitude, consistency, directness, and distance from medication safety. GRADE certainty was assigned by outcome using risk of bias, inconsistency, indirectness, imprecision, and publication bias; hybrid categories such as “low-moderate” were not used [17].

## 3. Results

### 3.1. Evidence Landscape

**Figure 3:**
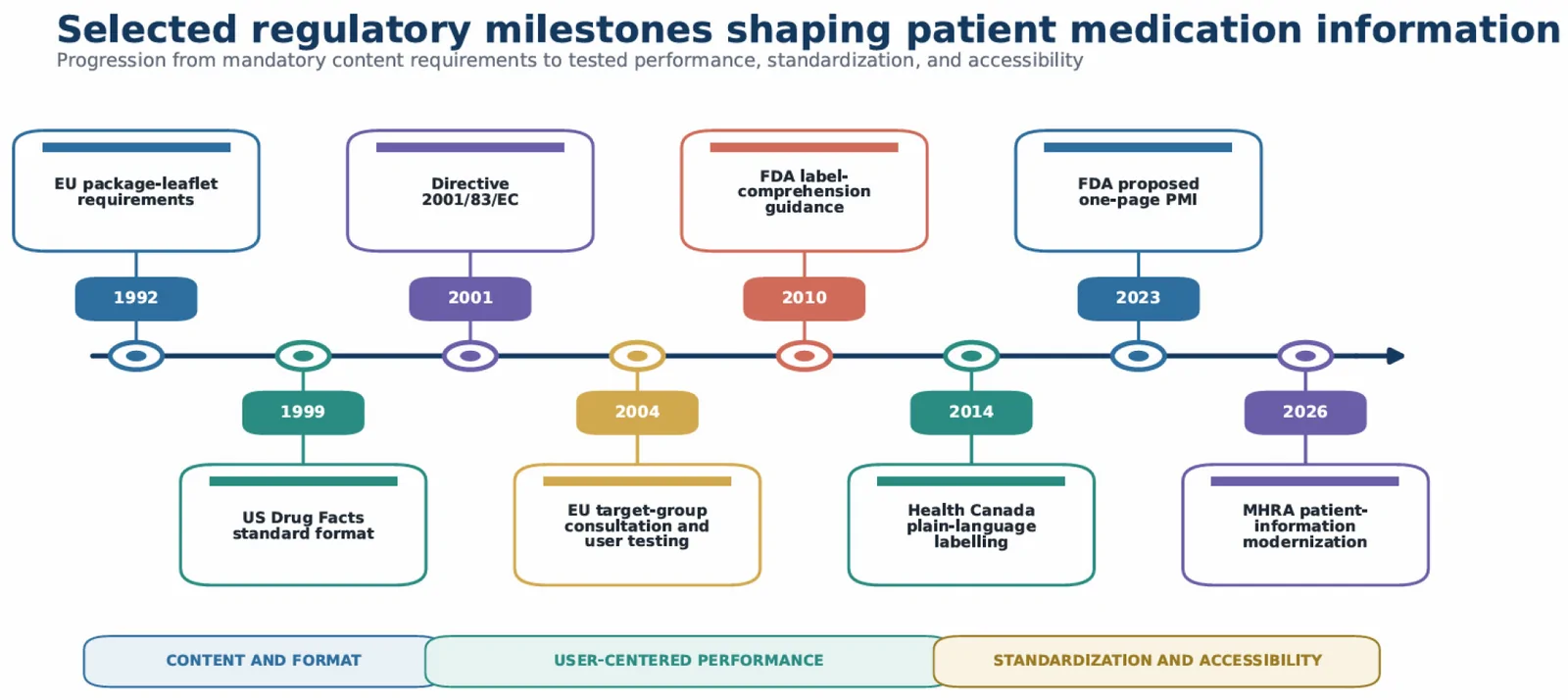
Selected regulatory milestones shaping patient medication information.

The included evidence spans the European Union, the United Kingdom, the Netherlands, Sweden, the United States, and Australia, with publication years from 2001 to 2024. The regulatory timeline in Figure 3 shows that empirical evaluation often followed major policy changes by several years. The evidence base was dominated by controlled communication experiments and user tests; fewer studies evaluated routine delivery or national implementation. Sample sizes ranged from 20-person diagnostic user tests to a probability-panel survey of 5204 US adults. Most experiments measured immediate information finding, comprehension, or risk perception. Only one randomized study moved beyond stated beliefs to symptoms attributed to a placebo, and no included study directly demonstrated a reduction in medication errors, adverse drug events, hospitalization, or mortality.

Supplementary Table S1 presents all 28 reports with jurisdiction, regulatory linkage, design, sample or unit, information product, intervention, comparator, outcomes, key findings, limitations, DOI, and source URL. One pair of companion reports used the same national survey sample and was counted as two reports but one study. This report-level table, together with the study-level appraisal table, makes the narrative synthesis independently auditable.

**Figure 4:**
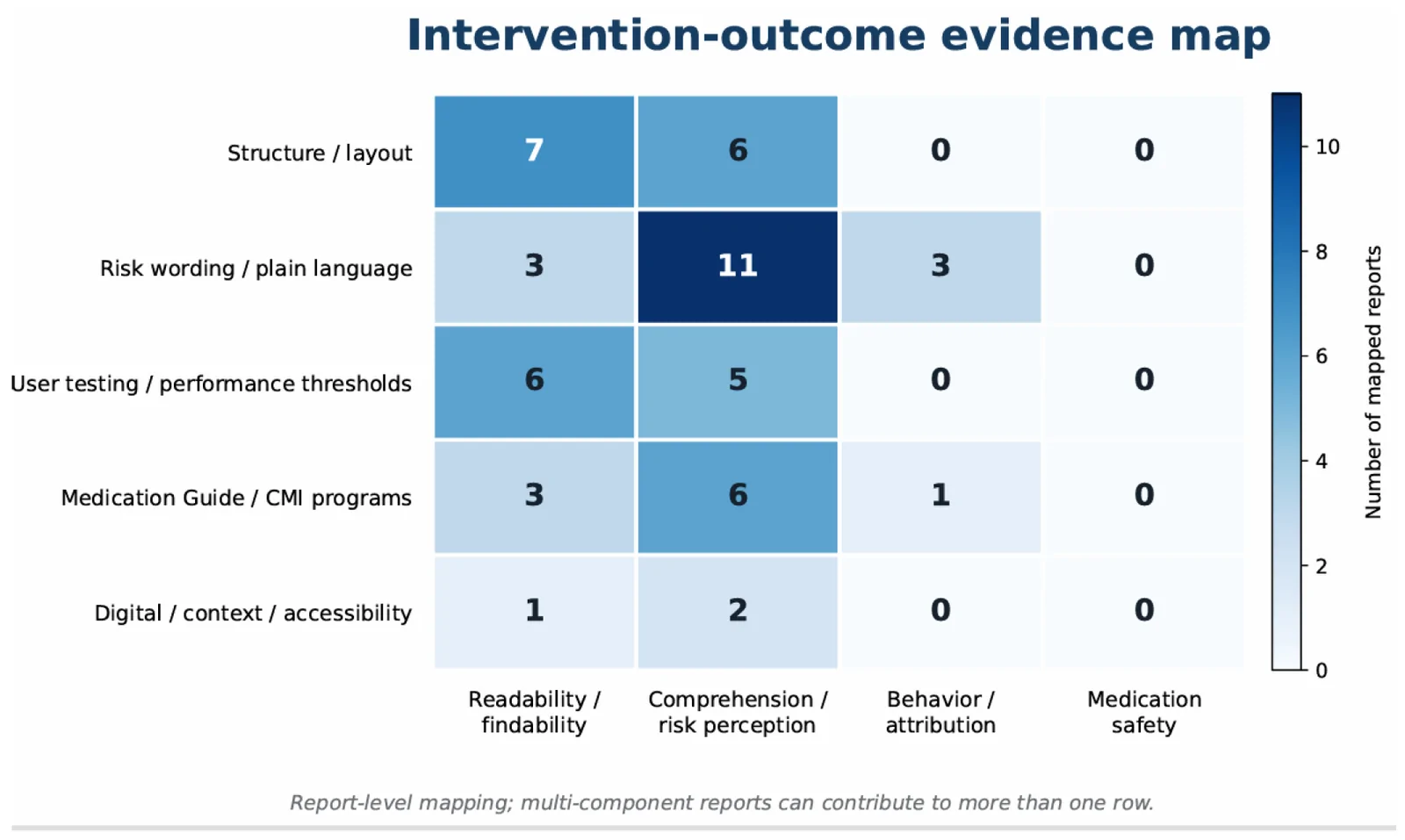
Intervention-outcome evidence map.

Four broad evidence clusters emerged. The first examined document structure, headings, layout, and user testing within the EU PIL system [18,19,20,21,22,23]. The second tested regulatory side-effect frequency wording and risk framing [24,25,26,27,28,29,30,31,32,33,34]. The third evaluated standardized US or Australian nonprescription labels using performance thresholds or controlled comparisons [35,36,37,38,39]. The fourth assessed Medication Guides, pharmacy CMI, and prototype PMI formats in the United States [40,41,42,43,44,45]. Several reports contributed to more than one cluster, particularly when a redesign combined structure, language, and testing. Figure 4 maps the density of evidence and makes the central gap visible: readability, findability, comprehension, and risk perception are well represented, whereas behavior and safety are not. The intervention clusters and principal evidence gaps are summarized in Table 2.

### 3.2. Standardization of Content, Structure, and Layout

The most direct test of a prescriptive model compared the European Commission leaflet design with an alternative based on information-design principles. In matched groups of 20 consumers, the prespecified threshold of 16 correct users was reached for only 3 of 15 key points with the EC model and for 8 of 15 with the alternative [18]. A larger sequential study of three EU-compliant leaflets found that users located only 75% of topics in the original versions; when they found the information, comprehension was approximately 90%. Redesign improved location success, speed, and comprehension [19]. These findings show that the main bottleneck may be navigation rather than decoding isolated sentences.

A bilingual experiment directly tested the mandatory EU template. One hundred twenty-eight participants used both current and revised structures. The revised real-text template produced a clear findability advantage (F = 14.22, *p* < 0.001, eta-squared = 0.101), and 68% of decisive respondents preferred it [20]. The intervention changed grouping, ordering, granularity, and heading wording. This makes mechanistic attribution difficult, but it also reflects real regulatory templates, which operate as systems rather than single typographic variables.

Headline sections illustrate why visual appeal and task performance should be measured separately. In an 80-person comparison, adding a headline section did not improve finding or understanding, although participants liked it [21]. In a later 20-person user test, the headline section was used in 39% of relevant searches and by 90% of participants at the beginning of testing [22]. The section may support orientation without replacing a coherent underlying structure. Similarly, Swedish evaluation of 30 PILs found generally sound information but poor comprehension of interactions and contraindications, especially when messages were complex and readers were older [23].

These results do not argue against standardization. A common structure can reduce search costs across products and make compliance easier to audit. Rather, the evidence favors a constrained-flexible model: regulators can mandate a prioritized core architecture while requiring topic grouping, headings, and navigation to be tested with intended readers. In this model, a template is an intervention to be evaluated, not a neutral administrative container.

### 3.3. Plain Language, Numerical Risk Communication, and Framing

The most consistent body of evidence concerns side-effect frequency descriptors. EU and EMA formats historically combined categories such as “very common,” “common,” “uncommon,” “rare,” and “very rare” with defined numerical ranges. Across four experiments involving more than 750 people, verbal descriptions produced substantial overestimation compared with quantitative information [26]. A related OTC scenario study found that recommended wording also affected intended action, showing that the problem is not restricted to abstract probability estimation [27].

The magnitude of distortion is clinically meaningful. In 120 patients taking statins, “common” produced a mean estimate of 34.2% compared with 8.1% for the numerical equivalent, while “rare” produced 18.0% compared with 2.1% [28]. Two randomized web experiments with 148 and 137 participants replicated higher estimates with verbal descriptors and suggested that natural frequencies can be more accurate than percentages for some readers [29]. In 187 participants, absolute frequencies improved accuracy for two of four side effects and overall estimates, increased satisfaction, and reduced perceived risk relative to verbal terms [30]. A 134-person factorial experiment similarly favored absolute frequencies over bands for two of four tamoxifen side effects and the overall probability [31].

Adding a verbal label to a numerical band does not solve the problem. In a 339-person trial, combined verbal-numerical expressions raised estimates for every assessed paclitaxel side effect, with four significant differences, whereas “may affect” and “will affect” had no material effect [32]. A national survey of 1003 English adults showed that 84.4% and 81.2% interpreted “very common” and “common” for mild effects as affecting more than 1 in 10, and adjacent verbal categories were poorly separated [24]. In a companion report, 51.9% considered “very common” and 45.0% considered “common” effects likely or very likely, compared with 8.1% for “uncommon,” 5.8% for “rare,” and 4.1% for “very rare” [25].

Risk framing can also alter experienced outcomes. In a double-blind placebo trial, 54.5% of participants given standard negative framing attributed symptoms to the tablet versus 39.2% given positive complementary framing; the odds ratio was 0.66 (95% CI 0.46–0.93) [33]. This does not establish a medication-safety benefit, but it demonstrates a plausible behavioral and nocebo pathway. An audit of 100 PILs found that only 66% of frequently dispensed products used the EMA-recommended format, so even a contested standard was incompletely implemented [34].

Regulatory language should therefore prioritize numerical information that preserves the denominator and makes the reference class explicit. Natural frequencies are often useful, but no single representation serves every task [13]. Testing should measure exact interpretation, comparative risk, recall, and intended action. Positive framing may be used as a complement when it remains balanced and does not hide harm. Verbal labels should not be allowed to dominate a numerical statement visually or semantically.

### 3.4. Performance-Based User Testing and Nonprescription Labels

Performance testing is a distinctive regulatory strategy because it converts communication into measurable objectives. The FDA naloxone Drug Facts program provides the largest example. Among 710 consumers, six of eight primary objectives met prespecified thresholds. Recognition of suspected overdose reached 95.8% (95% CI 94.0–97.1), and giving the first dose reached 98.2% (96.9–99.0). However, calling emergency services immediately reached 90.3% (87.9–92.4), and the composite sequence of checking, administering, and calling reached 81.1% (78.0–83.9) [35]. The label communicated individual actions better than the complete emergency sequence.

A pilot Drug Facts label for an over-the-counter combined oral contraceptive reached at least 95% on 10 of 11 primary and 10 of 11 secondary objectives among 163 participants. Important exceptions were a prior blood clot contraindication (89%) and recognition that the product applied to people able to become pregnant (83%) [36]. These residual gaps matter because a high average score can conceal failure on a small number of high-risk messages. Performance criteria should therefore weight criticality, not merely count objectives.

Australian user testing of four alternative standardized OTC label formats found satisfactory performance for all alternatives and weaker performance for the existing diclofenac label; the Consumer Desires and Drug Facts formats were most preferred [37]. Yet controlled US work found a narrower effect: Drug Facts improved scanning compared with scrambled prose but not cohesive prose, and did not improve recall, organization, or correction of misconceptions [38]. A separate comparison of old, standardized, and larger-font simulated labels favored larger font across ease of use and knowledge, suggesting that a six-point regulatory minimum may still be insufficient [39].

These findings favor iterative user testing, although they also show what such testing cannot establish. Interviews are usually conducted in quiet, supported settings and measure immediate performance. In routine use, purchasers may be ill, anxious, distracted, visually impaired, or choosing a medicine for someone else. Testing should therefore include lower-literacy and older adults, high-risk scenarios, realistic time pressure, and delayed or applied tasks. Regulators should identify “must-pass” safety messages in advance and require revision until both overall and subgroup thresholds are met.

### 3.5. Medication Guides, Consumer Medication Information, and Proposed PMI

Mandated information programs can fail at three separate stages: document design, delivery, and comprehension. An audit of 185 FDA-approved Medication Guides found a mean length of 1923 words (SD 1022), a reading level around grades 10–11, and only one guide rated suitable. In 449 patients, mean comprehension was 52.7% (SD 22.6); low literacy was associated with a 23.7-point lower score (95% CI −28.3 to −19.0) [40]. This is a large gap between regulatory approval and functional usability.

Pharmacy CMI showed a parallel implementation failure. Professional shoppers obtained lisinopril and metformin prescriptions at 365 pharmacies. Six percent received no written CMI. The material met only 60.2% and 57.7% of usefulness criteria on average, while comprehensibility and legibility scores were 43.8% and 42.6%. Only 3.0% of lisinopril and 0.3% of metformin materials met at least 80% of all criteria [41]. Distribution was common, but quality was neither standardized nor reliably useful.

Experimental simplification improved comprehension. In a randomized study of 1397 adults, one-page Bubbles and OTC-style prototypes both outperformed a four-page Medication Guide (*p* < 0.001), although applied decision performance did not differ and the two prototypes were similar [42]. An online experiment with 1119 adults found that additional qualitative context reduced side-effect comprehension, and that a hardcopy-like version combined with contextual text increased perceived risk [43]. More information is therefore not necessarily more informative; prioritization and architecture matter.

Regulatory assessments confirm that knowledge remains incomplete. Across 66 Medication Guide assessments submitted to FDA, mean primary-risk knowledge was 63.8%, and only 20 assessments (30.3%) met the 80% threshold. Programs containing additional REMS elements were more likely to reach that threshold, but survey methods varied substantially [44]. A later national probability-panel survey presents a more favorable perception: among 3852 adults who reported receiving a Medication Guide, 85% rated reading and 87% rated understanding from “about average” to “very easy” [45]. The contrast with objective studies is instructive. Perceived ease is a useful acceptability measure, but it cannot substitute for demonstrated comprehension.

The proposed US PMI policy addresses fragmentation by moving toward a standardized, concise document [8]. The empirical record supports that direction, provided implementation includes objective testing, pharmacy delivery verification, multilingual and accessible formats, and monitoring after launch. A single document should not be expected to carry the entire risk-minimization burden for medicines that require counseling, laboratory monitoring, restricted distribution, or shared decision-making.

### 3.6. Electronic Labeling, Accessibility, and Multilingual Information

Electronic labeling may ease some of the physical constraints of folded package inserts. Digital documents can support search, scalable type, audio, translation, hyperlinks, and rapid version updates. Access can still fail when it depends on a smartphone, connectivity, an unfamiliar authentication step, or a QR code separated from the medicine. Direct comparative evidence for electronic delivery was scarce in the included studies. In this area, policy development is currently ahead of evaluation.

The design principles supported by paper-based studies remain relevant online. Digital navigation should preserve a stable core hierarchy, make high-risk content reachable without scrolling through legal detail, and allow readers to switch between a concise overview and expanded evidence. Search functions cannot compensate for headings that do not match the user’s vocabulary. Dynamic content should carry a visible version date and retain an archive so that patients and clinicians can determine which warning applied at a given time. Regulators also need a defined fallback when digital access is unavailable.

Accessibility must be evaluated as performance, not as nominal compliance. Large type, screen-reader compatibility, contrast, keyboard navigation, audio, and plain-language translation should be tested with people who use those features. Multilingual information requires independent user testing because the bilingual EU template experiment showed that wording effects can vary by language even when the same architecture is used [20]. Literal translation of a poorly understood heading simply reproduces the problem. Cultural expectations about risk, medicine authority, and when to seek help may also alter interpretation.

On balance, the evidence favors a hybrid standard: a concise printed core document supplied with the medicine and an accessible digital version offering synchronized updates and optional detail. Both versions should use the same message hierarchy and section identifiers so that clinicians can direct patients to specific content. Evaluation should address successful access, time to reach critical information, comprehension, and disparities related to age, disability, language, and digital resources.

## 4. Discussion

This review shows a consistent gradient of evidence: interventions targeting structure, navigation, wording, and user testing improve proximal communication outcomes more reliably than medication behavior or clinical safety. The strongest and most reproducible findings were improved findability after user-centered redesign and reduced risk overestimation when numerical formats replaced verbal frequency descriptors. In contrast, formal approval, mandated distribution, or high overall test scores did not ensure that patients received the correct document, understood every critical message, or used medicines more safely. No included comparative study directly demonstrated fewer medication errors, adverse drug events, emergency visits, or hospitalizations. A 2015 systematic review focused primarily on the readability of medicinal package leaflets, while a 2021 scoping review catalogued leaflet and patient factors that facilitate or hinder comprehension [11,12]. The present synthesis extends that work by restricting inclusion to explicit regulator-linked interventions and by separating legal existence, delivery fidelity, findability, comprehension, behavior, and safety. It identifies where the regulatory chain most often breaks: information may be legally complete but poorly navigable, understandable under supported testing but not delivered in practice, or successful for immediate comprehension while untested for delayed behavior and safety. Readability, preference, and overall comprehension scores should therefore be treated as intermediate outcomes rather than proxies for clinical benefit.

### 4.1. Outcome Measurement and Harmonization

The studies also show why regulatory evaluation needs a common outcome architecture. Readability formulas describe surface features such as sentence and word length; they do not establish that a warning can be found, understood, or applied. Findability should be measured with realistic scenarios and time-to-location, while comprehension should separate recognition from unaided explanation. Risk communication requires calibrated numerical estimates rather than agreement with vague verbal categories. Applied tasks should ask participants to select a dose, identify a contraindication, sequence emergency actions, or decide when professional advice is required. Critical messages should be analyzed individually. The naloxone and oral-contraceptive studies achieved high overall performance while retaining important weaknesses in emergency sequencing, clot history, or product applicability [35,36]. An average score can therefore mask a clinically unacceptable failure. Regulators should specify a hierarchy in advance: life-threatening contraindications and emergency actions require a higher threshold than storage details, and performance should be reported with confidence intervals for each must-pass objective. Time also matters. Immediate interview performance may overestimate what a patient remembers at home. A core evaluation set should include immediate finding and understanding, delayed recall, and an applied task after a realistic interval. For mandated programs, delivery indicators should include the proportion of eligible dispensings with the correct document, opening or reading where measurable, version accuracy, and accessibility failures. Downstream studies should use objective adherence, dispensing changes, poison-center calls, medication errors, adverse-event reports, and healthcare use, with careful analysis of unintended effects such as avoidance of beneficial treatment. More consistent outcome reporting would make the evidence easier to accumulate. A small international core outcome set could retain local flexibility while allowing comparison across products and jurisdictions. It should record the information product, version, drug risk, participant literacy and language, exposure mode, outcome definition, denominator, time point, and whether the measure was objective or self-reported. This would support future meta-analysis and help distinguish transferable design principles from isolated successes under highly controlled conditions.

### 4.2. Effects on Behavior, Medication Safety, and Equity

Evidence weakens as outcomes move downstream. Most studies measured the ability to find an answer, select a response, estimate a probability, or report perceived ease. Positive framing reduced attribution of placebo symptoms [33], and risk wording influenced willingness or expectations in several experiments, but objective adherence and correct medicine use were rarely observed. No included comparative study directly estimated the effect of an information regulation on medication-error rates, overdose, preventable adverse drug events, emergency visits, or hospitalization. This gap does not mean patient information is unimportant. It means the regulatory evidence chain is incomplete. Proximal measures are necessary because a person cannot act correctly on information that cannot be found or understood. They are not sufficient because behavior depends on motivation, trust, prior beliefs, symptom burden, cost, clinician advice, and the feasibility of the recommended action. Safety outcomes are also uncommon and may require large linked datasets or interrupted time-series designs. Equity findings are more consistent. Objective Medication Guide comprehension was sharply lower among people with limited literacy [40], while the national survey found more favorable perceived ease among people with adequate health literacy but only modest model explanatory power [45]. Older participants had particular difficulty with complex interaction and contraindication messages [23]. Multilingual template work showed that wording effects can vary by language even when overall template effects are similar [20]. Digital access, disability, and visual acuity were seldom measured directly. Regulators should therefore require subgroup recruitment and report performance separately rather than treating an overall average as evidence of equitable communication.

### 4.3. Certainty of Evidence and Research Gaps

The certainty of evidence is Moderate for the conclusion that structure and user-centered revision improve findability under test conditions: effects were replicated, but interventions were often multi-component. Certainty is Moderate for the conclusion that verbal side-effect frequency labels inflate risk perception because the effect was large and experimentally replicated, although risk perception remains a surrogate. Certainty is Low for comprehension and knowledge because instruments, thresholds, and critical-item performance varied. Evidence for behavior is Low, implementation and equity evidence is Low, and medication-safety evidence is Very low because no direct comparative study estimated medication errors or adverse drug events. Table 3 summarizes the judgments; domain-level downgrading decisions are reported in Supplementary Table S3. Methodological limitations included convenience samples, incomplete concealment, sequential rather than concurrent comparisons, selective emphasis on successful items, and reliance on self-report. Most studies were not designed to estimate heterogeneous effects by literacy, language, disability, or age. Many tested fictional products or scenarios, which improves experimental control but reduces directness. Implementation studies often described whether material was provided or perceived as easy without verifying reading, exposure, or knowledge. Future evaluations should use pre-registered core outcomes, retain item-level data for critical messages, and link document exposure to dispensing, behavior, and clinical events. Cluster randomized implementation trials, stepped-wedge rollouts, controlled interrupted time series, and natural experiments can evaluate policy changes without withholding information. Common outcomes should include time to locate information, correct understanding of dose and contraindications, calibrated risk estimates, teach-back performance, objective correct-use tasks, dispensing coverage, medication errors, and preventable harm. Materials, code, and de-identified item-level data should be shared to permit independent verification and cross-jurisdiction comparison.

### 4.4. Regulatory and Practice Perspective

**Figure 5:**
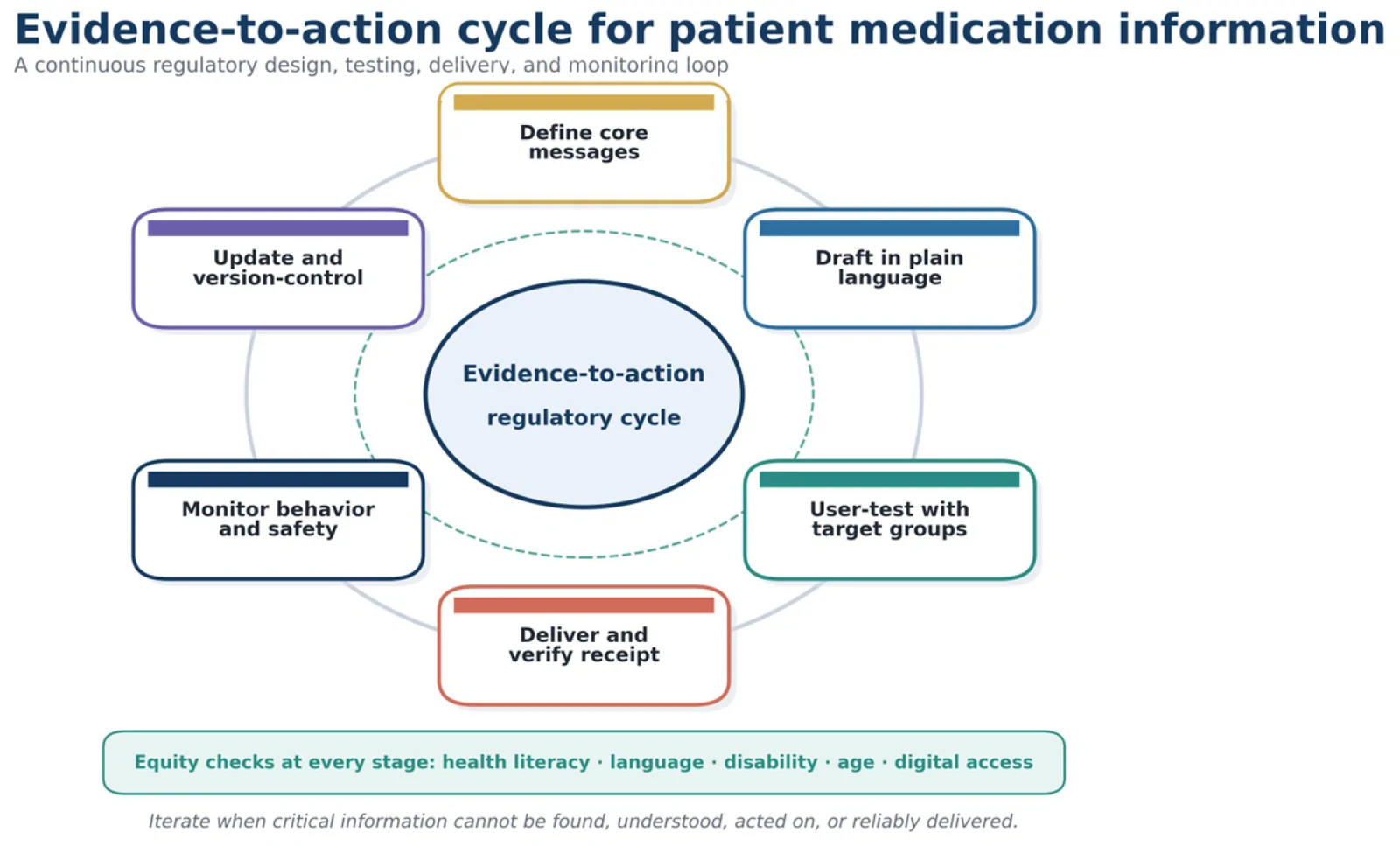
Evidence-to-action regulatory cycle for patient medication information.

Figure 5 converts the evidence into a six-stage cycle. First, regulators should define a small set of core messages and rank them by consequence if misunderstood. Second, developers should draft in plain language, use explicit headings and adequate typography, and present risks numerically with a clear denominator. Third, target users should complete realistic finding, understanding, and application tasks; critical items should have separate pass thresholds. Fourth, the system should verify that the correct version is actually delivered in print and accessible digital formats. Fifth, implementation should be monitored using coverage, complaints, knowledge, medication errors, and safety signals. Sixth, the information should be updated with version control and re-tested when risks or delivery channels change. A practical regulatory model lies between two extremes. Fully rigid templates can preserve structures that perform poorly, whereas unrestricted flexibility can create inconsistency and complicate oversight. The core sequence, safety hierarchy, minimum typography, accessibility requirements, and outcome thresholds should be fixed, with evidence-based adaptation allowed within sections. Manufacturers and information vendors should report design decisions and user-test results, including unsuccessful iterations. Pharmacists and clinicians should confirm receipt and understanding of high-risk messages, using teach-back when needed. Teach-back asks a patient to explain in their own words how they will take the medicine, what warning signs require action, or when to seek help; it can reveal a misunderstanding that a yes/no check or a favorable document-level score would miss and allows the clinician to correct the specific message. It is a practical bridge between document performance and safe use, especially for complex dosing, contraindications, and emergency instructions, but it requires time, training, and a clear core message set. Clinical communication should supplement rather than compensate for defective documents.

For clinical practice, the review supports a short, prioritized set of safety messages that pharmacists and clinicians can reinforce at dispensing or consultation, with teach-back focused on the highest-consequence misunderstandings. For policymakers, the findings support minimum content and accessibility standards, subgroup user testing, pharmacy delivery audits, version control, and post-implementation surveillance linked to dispensing and safety indicators. These measures make accountability extend beyond approval of the document to whether the intended person received, understood, and could apply the information.

### 4.5. Limitations

The review has the following limitations. The protocol was not prospectively registered, which increases concern about analytical flexibility despite prespecification before final screening. Dedicated grey-literature databases were not searched, and reports not indexed in the selected bibliographic sources or regulator websites may have been missed. Because reviewer-level screening decisions were not archived as 2 × 2 tables, Cohen’s kappa could not be calculated retrospectively; consensus procedures and the full-text exclusion log are provided instead. The evidence was highly heterogeneous, several reports provided limited methodological detail, and most outcomes were immediate communication surrogates rather than behavior or clinical safety. The tightened regulatory-linkage definition improves causal interpretability but excludes investigator-developed materials that may still inform design. These constraints precluded meta-analysis and limit causal inference about downstream medication outcomes.

## 5. Conclusions

Regulatory interventions can improve patient medication information, although the clearest effects occur early in the evidence chain. Standardized structures, information-design revision, concise formats, and target-user testing were generally associated with better information finding and understanding. Numerical risk formats performed more reliably than verbal frequency categories, which often led readers to overestimate side-effect probabilities. Findings from Medication Guide and CMI programs also show that formal approval or widespread distribution does not ensure readability, comprehension, or knowledge of primary risks.

Evidence was much thinner for medication-taking behavior and was nearly absent for real-world safety outcomes in routine practice. A better readability score or a successful interview should therefore not be interpreted as proof of fewer medication errors or adverse events. The evidence supports a narrower course of action: improve the core document, test its performance in diverse users, verify delivery, and measure downstream outcomes after implementation.

A sustainable policy would treat patient information as an iterative process: define and prioritize core messages, draft them in plain and accessible language, test them with target users, verify delivery, monitor outcomes, and update the material under version control. This retains the advantages of standardization while ensuring that key regulatory assumptions remain open to empirical testing.

## Figures and Tables

**Table 1:** Eligibility framework and operational definitions.

Operational Definition	Element
Patients, members of the public, caregivers, professionals, or patient-facing medicine information products.	Population/unit
An identifiable regulator-mandated requirement; regulator-endorsed guidance, template, or wording convention; standardized performance-testing requirement; mandated information program; or an intervention explicitly evaluating a named regulatory initiative.	Required regulatory linkage
Prior format, alternative regulatory wording or template, performance threshold, another regulatory program, or implementation standard.	Comparator
Readability/findability; comprehension/knowledge; risk perception; behavior; medication errors/adverse drug events; delivery, accessibility, and equity.	Outcomes
Randomized experiments, nonrandomized comparative tests, sequential user tests, label-comprehension studies, regulatory assessment reviews, implementation audits, and national surveys with explicit regulatory exposure.	Eligible designs
Investigator-developed information without named regulatory linkage; legislation without outcome data; purely descriptive readability audits; general health education; clinician communication training; non-medicine information sheets.	Key exclusions

**Table 2:** Intervention Clusters, Findings, and Evidence Gaps.

Principal Limitation or Gap	Most Consistent Finding	Intervention Cluster
Most evaluations used immediate tasks and multi-component redesigns.	User-centered redesign often improved information location, speed, and comprehension.	Structure, headings, and layout
Risk perception is a surrogate; behavioral and clinical consequences were rarely measured.	Verbal frequency descriptors consistently inflated perceived side-effect probability compared with numerical formats.	Risk wording and framing
Testing conditions were supported and may not represent real-world purchase or dispensing.	Critical information could be communicated at high overall rates, but must-pass items still failed.	Performance-based user testing
Delivery, exposure, and objective knowledge were inconsistently linked.	Formal approval or widespread distribution did not ensure readability, comprehension, or useful content.	Medication Guide and CMI programs
Digital access, disability, language, and version control were seldom evaluated.	Digital delivery offers search, scaling, audio, and updating, but direct comparative evidence was limited.	Digital and accessible information

**Table 3:** Certainty of Evidence by Outcome Domain.

Reason for Judgment	Certainty	Evidence Pattern	Outcome Domain
Downgraded one level for methodological limitations; effects replicated across settings.	Moderate	Multiple comparative redesign and user-testing reports	Findability/usability
Downgraded for risk of bias and inconsistency; critical-item failures remained despite favorable averages.	Low	Comparative experiments, label-comprehension studies, surveys	Comprehension/knowledge
Large, replicated effect; downgraded for indirectness to patient-important outcomes.	Moderate	Multiple randomized experiments plus national survey evidence	Risk perception
Two-level downgrade from experimental starting point for indirectness and imprecision.	Low	One placebo-attribution RCT plus intention/expectation outcomes	Behavior
No included comparative study estimated medication errors, preventable adverse drug events, emergency visits, hospitalization, or mortality.	Very low	No direct comparative evidence	Medication safety
Consistent descriptive gaps, but limited causal evidence and incomplete equity measurement.	Low	Document audits, delivery audits, comprehension and national surveys	Implementation/equity

Note: RCT, randomized controlled trial.

## Data Availability

The data supporting the findings of this review are included in the article and Supplementary Materials. Further inquiries can be directed to the corresponding author.

## Supplementary Materials

The following supporting information can be downloaded at https://www.mdpi.com/article/10.3390/healthcare14203464/s1. File S1: Final Executed Database Search Strategies and Search-Count Audit; File S2: PRISMA 2020 Reporting Checklist; Data S1: Evidence Tables and Appraisal Workbook, containing Table S1: Characteristics and Extracted Findings of Included Reports, Table S2: Design-Specific Methodological Appraisal, Table S3: GRADE Evidence Profile by Outcome Domain, Table S4: Full-Text Reports Excluded after Dual Review, PRISMA 2020 Count Reconciliation, Independent Dual-Reviewer Verification—Included Reports, and Near-Cutoff Eligibility Audit of Potentially Relevant 2025–2026 Records.

## Abbreviations

The following abbreviations are used in this manuscript:

PIL	Patient information leaflet
CMI	Consumer medication information
PMI	Patient medication information
PRISMA	Preferred Reporting Items for Systematic reviews and Meta-Analyses
ADEs	Adverse drug events
RCT	Randomized Controlled Trial
