# Supplementary material for: Regulatory Interventions for Package Inserts and Patient Medication Information: A Systematic Review
Source: Healthcare (Basel). 2026 Oct 15;14(20):3464. doi: 10.3390/healthcare14203464 (PMC13704687; doi:10.3390/healthcare14203464)
Supplement: Supplementary file 1 [file healthcare-14-03464-s001.zip › File S1.pdf]

Supplementary File S1. Final Executed Database Search Strategies and Search-Count Audit

Search date: July 23, 2026. Coverage: database inception to the search date. No date or language limits were applied during retrieval. Native counts were recorded before deduplication. The four database counts sum to 2,817 records.

Source	Platform/index	Native count
PubMed/MEDLINE	PubMed	612
Embase	Elsevier	1034
Web of Science Core Collection	Clarivate Core Collection	548
Scopus	Elsevier	623
Combined total	All four databases	2817

PubMed/MEDLINE (PubMed)

("Drug Labeling"[Mesh] OR "Patient Education Handout"[Mesh] OR package insert*[tiab] OR patient information leaflet*[tiab] OR medicine information leaflet*[tiab] OR medication guide*[tiab] OR consumer medicine information[tiab] OR consumer medication information[tiab] OR patient medication information[tiab] OR Drug Facts label*[tiab] OR prescription drug label*[tiab] OR patient-facing medicine information[tiab]) AND (regulat*[tiab] OR mandat*[tiab] OR standard*[tiab] OR guideline*[tiab] OR template*[tiab] OR format*[tiab] OR redesign*[tiab] OR "user testing"[tiab] OR usability[tiab] OR readability[tiab] OR comprehens*[tiab] OR "risk communication"[tiab] OR "plain language"[tiab] OR "label comprehension"[tiab] OR "information design"[tiab] OR implementation[tiab])

Embase (Elsevier)

('drug labeling'/exp OR 'patient education'/exp OR 'package insert*':ti,ab,kw OR 'patient information leaflet*':ti,ab,kw OR 'medicine information leaflet*':ti,ab,kw OR 'medication guide*':ti,ab,kw OR 'consumer medicine information':ti,ab,kw OR 'consumer medication information':ti,ab,kw OR 'patient medication information':ti,ab,kw OR 'drug facts label*':ti,ab,kw OR 'prescription drug label*':ti,ab,kw) AND ('regulation'/exp OR regulat*:ti,ab,kw OR mandat*:ti,ab,kw OR standard*:ti,ab,kw OR guideline*:ti,ab,kw OR template*:ti,ab,kw OR

format*:ti,ab,kw OR redesign*:ti,ab,kw OR 'user testing':ti,ab,kw OR usability:ti,ab,kw OR readability:ti,ab,kw OR comprehens*:ti,ab,kw OR 'risk communication':ti,ab,kw OR 'plain language':ti,ab,kw OR 'label comprehension':ti,ab,kw OR 'information design':ti,ab,kw OR implementation:ti,ab,kw)

Web of Science Core Collection (Clarivate)

TS=(("package insert*" OR "patient information leaflet*" OR "medicine information leaflet*" OR "medication guide*" OR "consumer medicine information" OR "consumer medication information" OR "patient medication information" OR "Drug Facts label*" OR "prescription drug label*") AND (regulat* OR mandat* OR standard* OR guideline* OR template* OR format* OR redesign* OR "user test*" OR usability OR readability OR comprehens* OR "risk communication" OR "plain language" OR "label comprehension" OR "information design" OR implementation))

Scopus (Elsevier)

TITLE-ABS-KEY(("package insert*" OR "patient information leaflet*" OR "medicine information leaflet*" OR "medication guide*" OR "consumer medicine information" OR "consumer medication information" OR "patient medication information" OR "Drug Facts label*" OR "prescription drug label*") AND (regulat* OR mandat* OR standard* OR guideline* OR template* OR format* OR redesign* OR "user test*" OR usability OR readability OR comprehens* OR "risk communication" OR "plain language" OR "label comprehension" OR "information design" OR implementation))

Supplementary sources

FDA, EMA/European Commission, MHRA, and Health Canada websites were checked for policy context and empirical reports. Backward and forward citation searching was performed from included reports and key reviews. These checks added no records beyond the database set used in the PRISMA counts.

Deduplication and count reconciliation

Sequential deduplication removed 637 records: 294 duplicates when Embase was added, 181 when Web of Science was added, and 162 when Scopus was added. This left 2,180 unique records. All 34 reports sought for full-text review were retrieved. Six were excluded, and 28 reports were included.
