# Supplementary material for: Regulatory Interventions for Package Inserts and Patient Medication Information: A Systematic Review
Source: Healthcare (Basel). 2026 Oct 15;14(20):3464. doi: 10.3390/healthcare14203464 (PMC13704687; doi:10.3390/healthcare14203464)
Supplement: Supplementary file 1 [file healthcare-14-03464-s001.zip › File S2.pdf]

Supplementary File S2. PRISMA 2020 Reporting Checklist

Item	Requirement (paraphrased)	Location in submission
1	Identify the report as a systematic review.	Title
2	Provide a structured summary of objectives, methods, results, and conclusions.	Summary and Abstract
3	Describe the rationale in the context of existing knowledge.	Introduction
4	State explicit review objectives.	End of Introduction
5	Specify eligibility criteria and synthesis grouping.	Review methodology - Eligibility criteria
6	Specify all information sources and last search date.	Review methodology - Information sources; Supplementary File 1
7	Present full search strategies.	Supplementary File 1
8	Describe independent selection and disagreement resolution.	Study selection, extraction, and independent verification
9	Describe data collection and verification.	Study selection, extraction, and independent verification

Item	Requirement (paraphrased)	Location in submission
10a-b	Define outcomes and other extracted variables.	Eligibility criteria; Supplementary Table S1
11	Describe study-level methodological appraisal.	Methodological appraisal, certainty, and synthesis; Supplementary Table S2
12	Define effect measures used in interpretation.	Methodological appraisal, certainty, and synthesis; narrative effect magnitudes
13a-f	Describe synthesis eligibility, preparation, tabulation, synthesis, heterogeneity, and sensitivity approach.	Methodological appraisal, certainty, and synthesis; Results; Discussion
14	Describe assessment of reporting bias.	Methodological appraisal, certainty, and synthesis; Discussion
15	Describe certainty assessment.	Methodological appraisal, certainty, and synthesis; Table 3; Supplementary Table S3
16a-b	Report selection results and cite excluded reports.	Figure 2; Supplementary Table S4

Item	Requirement (paraphrased)	Location in submission
17	Present characteristics of included studies.	Results; Supplementary Table S1
18	Present study-level methodological judgments.	Supplementary Table S2
19	Present results of individual studies.	Results (intervention-specific subsections); Supplementary Table S1
20a-d	Present synthesis results and heterogeneity/robustness considerations.	Results; Table 2; Figure 4
21	Present reporting-bias considerations.	Discussion
22	Present certainty of evidence.	Table 3; Supplementary Table S3
23a-d	Interpret findings, limitations, implications, and research needs.	Discussion; Conclusions
24a-c	Provide registration/protocol status and amendments.	Review design and reporting; protocol not prospectively registered
25	Describe support.	Funding
26	Declare competing interests.	Disclosures

Item	Requirement (paraphrased)	Location in submission
27	Describe availability of data, materials, and code.	Data Availability; Supplementary Data 1

Note: The checklist is a location map. Full methodological detail and report-level evidence are supplied in the cited manuscript sections and supplementary files.
